# Vancomycin‐induced linear IgA bullous dermatosis (LABD)—an atypical presentation

**DOI:** 10.1002/ccr3.2039

**Published:** 2019-04-22

**Authors:** Lorena Visentainer, Juliana Yumi Massuda, Maria Letícia Cintra, Renata Ferreira Magalhães

**Affiliations:** ^1^ State University of Campinas (UNICAMP) Campinas Brazil

**Keywords:** linear IgA bullous dermatosis, side effects of drugs, vancomycin, vesiculobullous disease

## Abstract

We report an uncommon presentation of bullous dermatosis by linear IgA. There are few cases reported in the literature with this form of presentation starting with mucosal lesions and then evolving into a similar bullous pemphigoid pattern. In addition, we emphasize the importance of direct immunofluorescence for the definitive diagnosis.

## 1 INTRODUCTION

A man presented with multiple blisters on the oral and genital mucosal after 10 days of administration of vancomycin. After this initial presentation, he started to present blisters in a symmetrical distribution. The whole investigation led to the diagnosis of linear IgA bullous dermatosis.

## 2 CASE REPORT

A 70‐year‐old man with a medical history of hypertension and diabetes was hospitalized to receive intravenous antibiotic due a surgical site infection. The infection started about 3 weeks after a surgical correction of an ascending thoracic aortic dissection. After 10 days of administration of vancomycin, the man presented with multiple tense blisters on oral and genital mucosal.

## 3 INVESTIGATIONS AND TREATMENT

**Figure 1:**
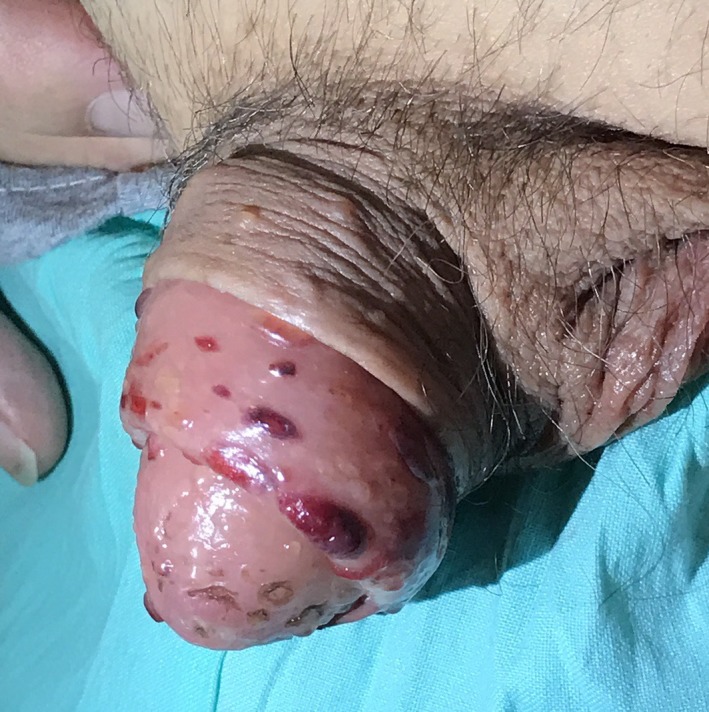
Multiple hemorrhagic vesicles on the glans

**Figure 2:**
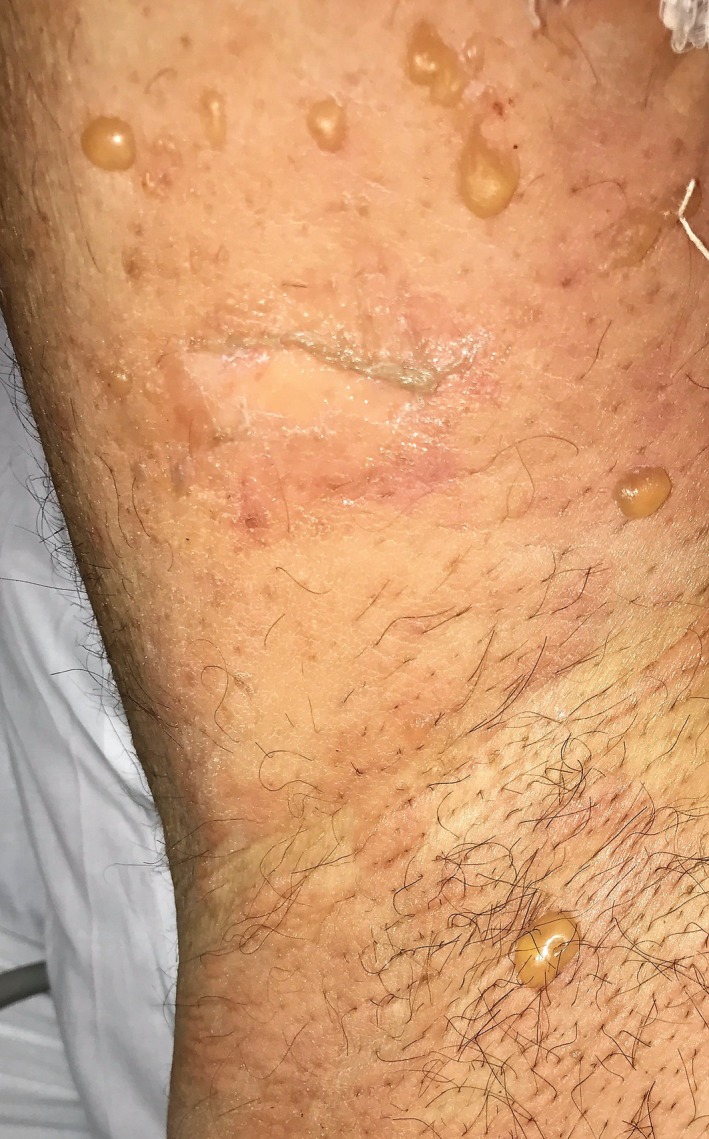
Fluid‐filled bullous lesions on an erythematous skin on axillary regions

Cutaneous examination showed multiple hemorrhagic vesicles on the soft palate and at the glans, neck, and shaft of the penis ranging from 1 to 4 cm in diameter (Figure 1). The initial presentation was like a herpetic infection, and we proceeded to a Tzanck smear from the roof of a vesicle which was negative to multinucleated giant cell. After 2 days, he started to present multiple bullous lesions fluid‐filled on an erythematous skin with a symmetrical distribution pattern on the inguinal and axillary regions (Figure 2). The clinical presentation was very similar to bullous pemphigoid. The Naranjo score^12^ for adverse drug reaction was 5 (probable drug reaction).

**Figure 3:**
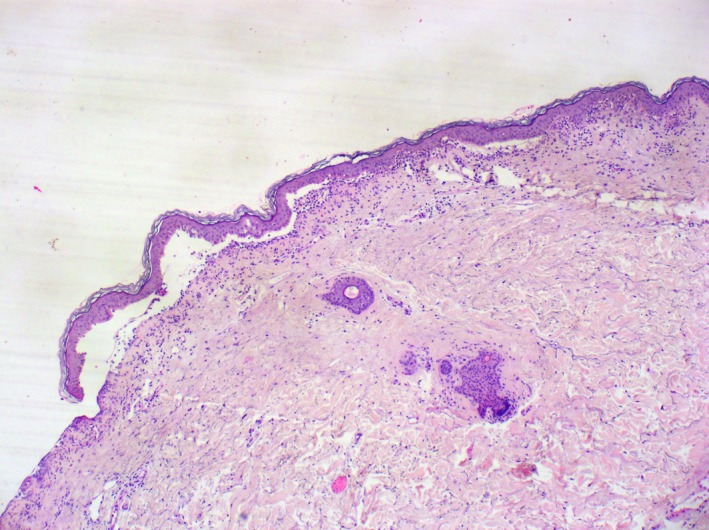
Histopathology showed subepidermal blister with inflammatory infiltrate (original magnification x40; hematoxylin‐eosin staining)

**Figure 4:**
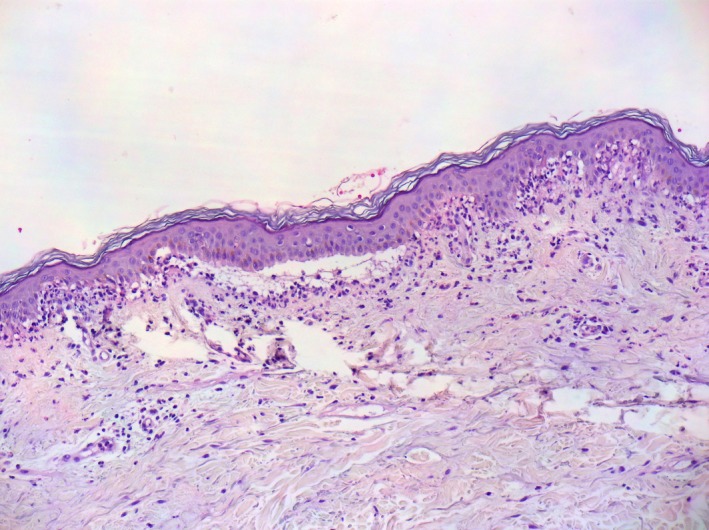
Subepidermal bulla containing neutrophilic infiltrate and adjacent papillary microabscesses (original magnification x100; hematoxylin‐eosin staining)

**Figure 5:**
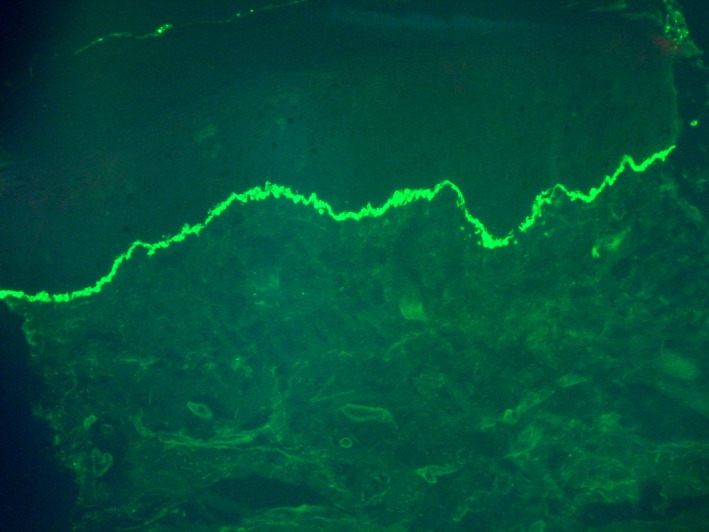
Direct immunofluorescence on perilesional skin revealed linear deposit of IgA along the dermo‐epidermal junction (original magnification x200)

Histopathology from a fresh vesicle showed subepidermal blister with neutrophilic and mixed inflammatory infiltrate (Figures 3 and 4). Direct immunofluorescence from study on perilesional skin revealed linear deposit of IgA along the dermo‐epidermal junction (Figure 5).

The only new medication identified at his prescription was vancomycin, which was suspended and replaced by another antibiotic class. Prednisone 1 mg/kg/day was introduced for 2 weeks and suspended after that, before we had the biopsy results. Then, he returned to the Dermatology clinic and he had complete resolution of his linear IgA bullous dermatosis at that time, and no additional drug was required.

## 4 OUTCOME AND FOLLOW‐UP

In our reported case, the clinical presentation was very atypical with mucosal manifestations which are rare in LABD and absence of annular erythema and blisters (often referred to as a “crown of jewels”) which are typical of the disease. We only could confirm the diagnosis of LABD due to the direct immunofluorescence, reinforcing its importance.

## 5 DISCUSSION

Linear IgA bullous dermatosis (LABD), also known as linear IgA dermatosis or linear IgA disease, is a rare subepidermal vesiculobullous disease that can occur in both adults and children. It is characterized by a linear deposition of IgA along the blister base, with a predominantly neutrophilic dermal infiltrate.^5^, ^6^, ^11^, ^13^, ^16^ LABD usually presents with tense blisters in a pearl necklace‐like arrangement and polycyclic urticarial plaques on the trunk and extremities.^8^, ^10^, ^16^ The immunofluorescence of perilesional skin is the gold standard to confirm the diagnosis.^3^, ^14^

In adult population, it may occur in two peaks, one in the teenage and early adult years, and the other in the sixth decade of life. In adults, it is commonly induced by drugs and the main drug related to LABD is vancomycin.^1^, ^2^, ^4^, ^7^, ^13^, ^16^ Vancomycin‐induced LABD is associated with a severe presentation of the disease that may mimic toxic epidermal necrolysis (TEN).^1^, ^5^, ^9^, ^15^ There have also been numerous reports of LABD mimicking erythema multiforme and TEN but not many reports of this disease with mucosal presentation similar to a herpetic infection and evolving to a bullous pemphigoid‐like clinic presentation. Multiple different treatment options have arisen for LABD but the most commonly used medication is oral dapsone, and it is essential to suspend the probable trigger drug.^1^

## CONFLICT OF INTEREST

None declared.

## AUTHOR CONTRIBUTION

LV: collection of data, performed the literature search, drafted the article, and photographed the patient. JYM: managed the case and critical revision of the article. MLC: involved in Histopathological and immunofluorescence images. RFM: involved in conception or design of the work and critical revision of the article. All authors contributed to the final version of the manuscript.
